# Risk Factors for Respiratory Failure in Patients Hospitalized With Systemic Sclerosis: An Analysis of the National Inpatient Sample

**DOI:** 10.7759/cureus.35797

**Published:** 2023-03-05

**Authors:** Amy Trang, Soumyasri Kambhatla, Augustine Manadan

**Affiliations:** 1 Internal Medicine, Rush University Medical Center, Chicago, USA; 2 Rheumatology, University of Illinois at Chicago, Chicago, USA; 3 Rheumatology, Rush University Medical Center, Chicago, USA

**Keywords:** hospitalizations, interstitial lung disease, systemic sclerosis, scleroderma, respiratory failure

## Abstract

Background

Systemic sclerosis (SSc) patients are at high risk for respiratory failure due to the progression of their disease. Investigating factors predictive of impending respiratory failure in this patient population can be used to improve hospital outcomes. Here, we investigate risk factors associated with developing respiratory failure in patients hospitalized with a diagnosis of SSc in the United States using a large, multi-year, population-based dataset.

Methodology

This retrospective study was conducted on SSc hospitalizations from 2016 to 2019 with and without a principal diagnosis of respiratory failure from the United States National Inpatient Sample database. A multivariate logistic regression analysis was performed to calculate adjusted odds ratios (OR_adj_) for respiratory failure.

Results

There were 3,930 SSc hospitalizations with a principal diagnosis of respiratory failure and 94,910 SSc hospitalizations without a diagnosis of respiratory failure. Among SSc hospitalizations, multivariable analysis showed that the following were associated with a principal diagnosis of respiratory failure: Charlson comorbidity index (OR_adj_ = 1.05), heart failure (OR_adj_ = 1.81), interstitial lung disease (ILD) (OR_adj_ = 3.62), pneumonia (OR_adj_ = 3.40), pulmonary hypertension (OR_adj_ = 3.59), and smoking (OR_adj_ = 1.42).

Conclusions

This analysis represents the largest sample to date in assessing risk factors for respiratory failure among SSc inpatients. Charlson comorbidity index, heart failure, ILD, pulmonary hypertension, smoking, and pneumonia were associated with higher odds of inpatient respiratory failure. Patients with respiratory failure had higher in-hospital mortality compared to those without it. Outpatient optimization and inpatient recognition of these risk factors can lead to improved hospitalization outcomes for SSc patients.

## Introduction

Scleroderma broadly encompasses two groups, namely, localized scleroderma, which only involves the skin, and systemic sclerosis (SSc), which has multiorgan system involvement [1]. This paper will discuss SSc, which affects about 25.9 per 100,000 people in the United States [2]. Although often recognized by skin involvement, SSc is a spectrum categorized into the following three subtypes: scleroderma sine scleroderma, limited scleroderma, and diffuse scleroderma. Scleroderma sine scleroderma has internal organ manifestations without obvious skin fibrosis. Limited scleroderma may have thickened skin distal to the elbows and knees, sometimes involving the face. In diffuse scleroderma, skin fibrosis may involve the proximal extremities and trunk [1]. The disease may lead to internal organ dysfunction with pulmonary, cardiac, and renal complications. The survival rate of SSc patients from the time of diagnosis has been estimated to be 62.5% at 10 years [3]. Although scleroderma renal crisis was previously identified as the leading cause of death in these patients, studies now show a shift to pulmonary complications [4].

A frequently recognized pulmonary manifestation of SSc is interstitial lung disease (ILD), which causes a decline in lung function and shows fibrosis on imaging [5]. The pattern of ILD progression and its complications in SSc are not easily understood as the clinical course may vary [5]. As such, the development of respiratory failure in SSc patients is not well studied for similar reasons. Our article investigates risk factors for respiratory failure in patients hospitalized with a diagnosis of SSc in the United States using a large, multi-year, population-based dataset.

## Materials and methods

Methods

Data Source

This is a retrospective study based on the data abstracted from the National Inpatient Sample (NIS) database (available online at http://www.hcup-us.ahrq.gov). This database is the largest collection of inpatient admission data in the United States. It is a nationally representative sample of 20% of hospitalizations from approximately 1,000 hospitals. The NIS was searched for hospitalizations in 2016, 2017, 2018, and 2019. Diagnoses for each hospitalization were recorded utilizing the International Classification of Diseases, Tenth Revision, Clinical Modification (ICD-10). In the NIS, diagnoses are divided into a principal diagnosis and one or more secondary diagnoses wherein the principal diagnosis is the main reason for the hospitalization. Secondary diagnoses are any ICD-10 codes other than the principal diagnosis. We did not seek institutional review board permission as all NIS data are de-identified and publicly available.

Inclusion Criteria and Study Variables

Our study group included all adult patients who were hospitalized with a diagnosis of SSc (M34 codes) with and without a principal diagnosis of respiratory failure (J96 codes). Race, gender, age, length of stay (LOS), total hospital charges, comorbidities, mean household income by zip code, and mortality were reported for the two groups. SSc patients with a secondary diagnosis of respiratory failure were excluded from this analysis because we aimed to analyze those hospitalized primarily for acute respiratory failure or acute-on-chronic respiratory failure. We used the following ICD-10 codes to identify comorbidities known to increase the risk for respiratory failure: heart failure codes I50 and I11.0; ILD codes J84.1; pneumonia codes J12-J18; pulmonary hypertension codes I27.2; and smoker/nicotine codes Z87.891, F17, and Z72.0.

Outcomes

The primary outcome was the identification of variables associated with a principal diagnosis of respiratory failure among SSc hospitalizations. The secondary outcomes were a description of demographic characteristics, total hospital charges, LOS, and mortality among SSc hospitalizations with and without respiratory failure.

Statistical analysis

We used Stata, version 16.1 (StataCorp, College Station, TX, USA) to perform the analyses. A univariable logistic regression analysis was used to calculate unadjusted odds ratios (ORs) for a principal diagnosis of respiratory failure. All variables with p-values ≤0.2 were included in a multivariable logistic regression model. Adjusted OR (OR_adj_) were reported and considered significant when p-values were <0.05. The Charlson Comorbidity Index (CCI) was used to adjust for comorbidity burden. Economic status was adjusted by income quartile (Q) of the patient’s home zip code. Q1 median household income was $1-42,999, Q2 median household was income $43,000-53,999, Q3 median household income was $54,000-70,999, and Q4 median household income was >$71,000.

## Results

There were 121,099,120 adult hospital discharges in the combined 2016-2019 NIS database. Of these, 3,930 had SSc and a principal diagnosis of respiratory failure. On the other hand, 94,910 hospitalizations had a diagnosis of SSc without a diagnosis of respiratory failure. Characteristics of respiratory failure and non-respiratory failure hospitalizations are displayed in Table 1.

**Table 1:** Weighted descriptive characteristics of adult SSc hospitalizations from 2016 to 2019 using the National Inpatient Sample database. CCI = Charlson Comorbidity Index; ILD = interstitial lung disease; HTN = hypertension; IQR = interquartile range; n = number; PI = Pacific Islander; SSc = systemic sclerosis

Hospitalization characteristics	SSC without respiratory failure (n = 94,910)	SSC with respiratory failure (n = 3,930)	P-value
Age, median (IQR)	64 (54-73)	63 (54-72)	0.869
Age groups (n, %)			
18–40 years	7,865 (8.3%)	160 (4%)	<0.001
40–60 years	28,235 (29.7%)	1,385 (35.2%)	0.001
60–80 years	47,760 (50.3%)	1,970 (50%)	0.918
>80 years	11,050 (11.6%)	415 (10.6%)	0.358
Women, (n, %)	80,395 (84.7%)	3,205 (81.6%)	0.019
Race (%)			
White	65.2%	60.6%	0.009
African American	14.8%	20.7%	<0.001
Hispanic	11.5%	9.7%	0.127
Asian or PI	2%	1.7%	0.516
Native American	0.9%	0.9%	0.911
Other race	2.5%	2.4%	0.852
Length of stay, mean days	5.2	7.7	<0.001
Total charges, mean	$59,257	$95,282	<0.001
CCI, mean	2.9	3.4	<0.001
Household income Q1 (%)	25%	27%	0.118
Household income Q2 (%)	25%	26%	0.430
Household income Q3 (%)	25%	24%	0.495
Household income Q4 (%)	24%	21%	0.059
Heart failure	21,660 (22.8%)	1,880 (47.8%)	<0.001
ILD	6,620 (7%)	1,105 (28%)	<0.001
Pneumonia, viral or bacterial (n, %)	7,915 (8.3%)	1,050 (26.7%)	<0.001
Pulmonary embolus (n, %)	1,220 (1.3%)	100 (2.5%)	0.003
Pulmonary HTN (n, %)	19,945 (21%)	2,265 (57.6%)	<0.001
Smoker (n, %)	30,830 (32.5%)	1,525 (38.8%)	<0.001
In-hospital death (n, %)	1,425 (1.5%)	570 (14.5%)	<0.001

Hospitalizations with respiratory failure versus those without respiratory failure had similar ages (median age 63 vs. 64; p = 0.869), were less likely to be female (81.6% vs. 84.7%; p = 0.019), were less likely to be white (60.6% vs. 65.2%; p = 0.009), were more likely to be African American (20.7% vs. 14.8%; p < 0.001), had a greater mean LOS (7.7 vs. 5.2 days; p < 0.001), had greater mean total hospital charges ($95,282 vs. $59,257; p < 0.001), had higher mean CCI (3.4 vs. 2.9; p < 0.001), and had higher in-hospital mortality (14.5% vs. 1.5%; p < 0.001). At p ≤0.2, the univariable analysis showed that CCI, gender, white, African American, Hispanic, income Q1, income Q4, heart failure, ILD, pulmonary embolus, pulmonary hypertension, smoking status, and pneumonia were associated with a principal diagnosis of respiratory failure (Table 2).

**Table 2:** Univariable analysis of predictors of respiratory failure in SSc hospitalizations. CCI = Charlson Comorbidity Index; ILD = interstitial lung disease; HTN = hypertension; PI = Pacific Islander; SSc = systemic sclerosis

Variable	OR	P-value	95% CI
Age	1.00	0.293	0.998-1.007
CCI	1.14	<0.001	1.103-1.169
Female	0.80	0.019	0.660-0.963
White	0.82	0.009	0.704-0.951
African American	1.50	<0.001	1.248-1.804
Hispanic	0.82	0.128	0.641-1.058
Asian or PI	0.83	0.517	0.478-1.450
Native American	1.04	0.911	0.489-2.227
Other race	0.96	0.852	0.603-1.519
Household income Q1	1.14	0.118	0.967-1.341
Household income Q2	1.07	0.430	0.904-1.267
Household income Q3	0.94	0.495	0.792-1.120
Household income Q4	0.84	0.059	0.702-1.007
Heart failure	3.10	<0.001	2.688-3.578
ILD	5.22	<0.001	4.418-6.161
Pneumonia, viral or bacterial	4.01	<0.001	3.392-4.734
Pulmonary embolus	2.01	0.003	1.259-3.192
Pulmonary HTN	5.11	<0.001	4.409-5.929
Smoker	1.32	<0.001	1.136-1.529

Among SSc hospitalizations, multivariable analysis showed that the following were associated with a principal diagnosis of respiratory failure: CCI (OR_adj_ = 1.05; 95% CI = 1.008-1.091), heart failure (OR_adj_ = 1.81; 95% CI = 1.520-2.153 ), ILD (OR_adj_ = 3.62; 95% CI = 3.015-4.341), pneumonia (OR_adj_ = 3.40; 95% CI = 2.837-4.081), pulmonary hypertension (OR_adj_ = 3.59; 95% CI = 3.037-4.233), and smoking (OR_adj_ = 1.42; 95% CI = 1.206-1.661) (Table 3).

**Table 3:** Multivariable analysis of predictors of respiratory failure in SSc hospitalizations. CCI = Charlson Comorbidity Index; ILD = interstitial lung disease; HTN = hypertension; SSc = systemic sclerosis

Variable	OR	P-value	95% CI
CCI	1.05	0.018	1.008-1.091
Female	0.88	0.227	0.718-1.082
White	0.99	0.967	0.766-1.291
African American	1.14	0.405	0.841-1.536
Hispanic	0.84	0.320	0.594-1.185
Household income Q1	0.97	0.785	0.809-1.174
Household income Q4	0.89	0.245	0.728-1.084
Heart failure	1.81	<0.001	1.520-2.153
ILD	3.62	<0.001	3.015-4.341
Pneumonia, viral or bacterial	3.40	<0.001	2.837-4.081
Pulmonary embolus	1.38	0.203	0.839-2.281
Pulmonary HTN	3.59	<0.001	3.037-4.233
Smoker	1.42	<0.001	1.206-1.661

## Discussion

Studies show a shift in the leading cause of mortality in SSc from renal crisis to pulmonary complications over the last four decades. In evaluating the Pittsburgh Scleroderma Databank, Steen et al. discovered that deaths related to renal crisis decreased from 42% to 6%, and deaths related to pulmonary fibrosis increased from 6% to 33% [6]. Similarly, a multinational study using the European League Against Rheumatism (EULAR) Scleroderma Trial and Research (EUSTAR) database found that pulmonary fibrosis was the leading cause of mortality in SSc at 35% of total disease-related deaths [4]. This study aimed to investigate risk factors associated with respiratory failure among SSc hospitalizations. In our study, 3,930 patients with SSc were hospitalized with a principal diagnosis of respiratory failure from 2016 to 2019 in the United States. Multivariable analysis showed that CCI, heart failure, ILD, pneumonia, pulmonary hypertension, and smoking were associated with respiratory failure. The risk for inpatient mortality in those with respiratory failure was 14.5%, comparable to a previous study showcasing that 11.98% of all in-hospital SSc deaths were due to respiratory failure [7].

Rheumatic disease patients are usually considered immunosuppressed due to their underlying, malfunctioning immune system, which sometimes can be further exacerbated by the treatments they receive. A multicenter study in France found that the mortality rate of ILD patients with coexisting systemic rheumatic disease admitted to the intensive care unit was 40% compared to 16% of rheumatic disease patients without ILD [8]. With the high mortality rate in this patient population, any identifiable risk factors leading to respiratory failure and the need for intensive care unit-level care should be carefully addressed and managed in both inpatient and outpatient settings. Our findings are crucial to keep in mind when caring for SSc patients, as recognition of such risk factors ahead of time can improve the quality of care and patient outcomes.

In review, ILD was the strongest predictor of respiratory failure in our analysis. The disease course of ILD ranges from mild, stable without treatment, to progressive requiring treatment [1]. In those with progressive disease, the rate of decline may differ; some have worsening symptoms in one year while others progress up to five years after follow-up [6,9]. Pulmonary hypertension can develop as a consequence of ILD or may be the result of a primary process driving vascular narrowing, leading to pulmonary arterial hypertension (PAH) [1]. Published in 2013, the DETECT algorithm aims to detect risks of developing PAH in SSc patients. The two-step process utilizes pulmonary function tests (PFTs), stigmata on physical examination, electrocardiography findings, labs, and echocardiogram to determine the need for diagnostic right heart catheterization in patients at risk for PAH [10]. Until 2020, there were no definitive guidelines for the diagnosis and management of ILD in SSc patients. The European consensus recently published an algorithm to include high-resolution CT (HRCT) and PFTs for ILD screening. As pulmonary fibrosis surpasses other causes of mortality in SSc patients, there is also ongoing research on therapeutic options for ILD. Cyclophosphamide (CYC) has been one of the oldest recommendations from the European guidelines for progressive lung disease in SSc-ILD [11]. Another therapy commonly used includes mycophenolate mofetil (MMF), for which the Scleroderma Lung Study II (SLS-II) found that MMF was better tolerated and had less toxicity than CYC; however, both led to improvement in breathing, lung function, imaging, and skin manifestations [12]. Rituximab was studied in the EUSTAR which improved skin fibrosis and prevented further lung fibrosis [13]. Recent studies evaluated the FDA-approved tyrosine kinase inhibitor, nintedanib, as well as tocilizumab to address pulmonary function decline with ILD [9,14]. Methotrexate was found to improve skin findings, with little to no effect on other organ manifestations such as ILD [15,16]. Hemopoietic stem-cell transplantation was also studied in severe SSc patients, showing improvement in skin and pulmonary function [17,18]. Potential treatment strategies for SSc-ILD continue to evolve with exciting advancements underway. Close outpatient screening with HRCT and PFTs as well as optimizing various treatment strategies for ILD in SSc patients is imperative to decreasing the risk of respiratory compromise in the hospital.

It was not surprising that pneumonia had three-fold increased odds for respiratory failure. In the EUSTAR cohort, pneumonia was the most common type of infection-related death [4]. Infectious pneumonia in SSc patients may be explained by several factors. The use of immunosuppressive treatments in this population increases the risk for both typical and opportunistic infections [19]. Esophageal dysmotility as a complication of SSc may lead to pulmonary aspirations, increasing pneumonia risk [20]. Proton pump inhibitors (PPIs) for acid reflux control in patients also contribute to increased rates of pneumonia. A meta-analysis by Lambert et al. found more than half of the 26 studies evaluated showed a greater risk of community-acquired pneumonia with PPI use [21]. Some studies postulate that acute bacterial pneumonia causing alveolar injury in a susceptible host with ILD may lead to the progression of pulmonary fibrosis [22].

Heart failure is a recognized complication of SSc and leads to increased rates of death, particularly in those with diastolic dysfunction or pulmonary hypertension complicated by left heart disease [19,23,24]. Our findings show that both heart failure and pulmonary hypertension substantially increased the odds of respiratory failure among SSc patients. SSc causes PAH as a consequence of microvascular damage, which leads to right ventricular (RV) dysfunction [25]. The prevalence of heart failure in SSc is about 20-25% with various proposed mechanisms of myocardial injury [26]. One theory is that fibrosis of the myocardium leads to both systolic and diastolic dysfunction [27]. Another observation is that RV overload affects the interventricular septum and ultimately impairs left ventricular (LV) function [28]. Other studies have discussed myocarditis, coronary artery disease, and arteriolar endothelial injury as possible etiologies of heart failure in SSc patients [26-29]. The association between heart failure and respiratory failure has been previously cited in the literature, sometimes presenting with impaired LV function leading to pulmonary edema [30,31,32]. Our findings highlight the need for close outpatient follow-up and management of heart failure in SSc patients to minimize inpatient respiratory decompensation.

Currently, both US and European guidelines recommend screening for PAH in SSc patients, which sometimes includes Doppler echocardiography to assess ventricular dysfunction [10,33]. Other studies discuss the utility of cardiac MRI (cMRI) in patients without cardiac symptoms and normal echocardiography results [26,34]. These investigators found that cMRI detected myocardial damage in as many as 75% of SSc cases evaluated [35]. Either through echocardiography or cMRI, early detection of cardiac dysfunction in relation to heart failure and/or PAH in SSc with multidisciplinary care among rheumatologists, pulmonologists, and cardiologists improves overall care for the patient and can potentially improve in-hospital outcomes.

Lastly, the current literature shows a high incidence of the female sex in SSc-ILD patients, ranging from 69% to 78% [2,36]. Our findings confirm that over 80% of patients hospitalized with a diagnosis of SSc were female, but the female sex was not a predictor of inpatient respiratory failure after adjustment for other demographic variables and medical comorbidities.

SSc is a rare disease, for which research involving large sample sizes is difficult to come by. Our study provides information on the associations between respiratory compromise and baseline characteristics, hospital costs, and multiorgan system effects of hospitalized SSc on a national level. We adjusted the outcomes for relevant comorbidities and demographic variables and produced adjusted odds ratios to better understand prevalent clinical patterns in these hospitalizations.

Limitations of our study include the potential for misdiagnoses in the NIS database, as ICD codes serve more for billing purposes than clinical use [37]. ICD-10 codes were used to identify our patient population, and it was not possible to review the chart to verify each SSc diagnosis. It should be mentioned, however, that the ICD code for SSc was validated according to the ACR/EULAR criteria in a study conducted in 2015 [38]. Another limitation of the NIS database was the lack of important information such as the type of therapy or immunosuppressive medication patients may have been on or baseline respiratory status such as home oxygen needs. Future studies may look into the chronicity of respiratory failure, as those with acute-on-chronic respiratory failure can have varying associations than those with only acute or chronic respiratory failure. Moreover, we could not differentiate between the various types of SSc, which could have been a predictive factor of respiratory decompensation [39]. Lastly, despite the inherent advantages of using a large dataset such as the NIS, a recognizable disadvantage is that hospital admissions marked as separate encounters could potentially be a duplicate if someone was readmitted within that same calendar year.

## Conclusions

This analysis represents the largest sample to date in assessing risk factors for respiratory failure among SSc inpatients. Patients with respiratory failure had higher in-hospital mortality compared to those without it. We identified CCI, heart failure, ILD, pulmonary hypertension, smoking, and pneumonia to be associated with higher odds of inpatient respiratory failure. Incorporating these variables in discussions with patients may assist with their understanding of the disease and the risks associated with potential inpatient respiratory failure. Optimizing outpatient multidisciplinary care in this multisystem disease can minimize inpatient decompensation and improve hospitalization outcomes for SSc patients.
